# Prognostic value and management of regional lymph nodes in locoregional breast cancer recurrence: a systematic review of the literature

**DOI:** 10.1007/s00404-021-06352-9

**Published:** 2022-02-04

**Authors:** Isabell Ge, Thalia Erbes, Ingolf Juhasz-Böss

**Affiliations:** 1 Department of Obstetrics and Gynecology, Medical Center - University of Freiburg, Freiburg, Germany; 2 Faculty of Medicine, University of Freiburg, Freiburg, Germany

**Keywords:** Breast cancer recurrence, Nodal status, Radiologic diagnosis, Prognosis, Repeat sentinel node biopsy, Aberrant drainage

## Abstract

**Purpose:**

Management of regional lymph nodes in breast cancer recurrence has been heterogeneous. To facilitate clinical practice, this review aims to give an overview on the prognosis, staging and operative management of (inapparent) regional lymph nodes.

**Methods:**

Current national and international guidelines are reviewed and a structured search of the literature between Jan 1, 1999 and Feb 1, 2021 on the repeat sentinel node biopsy (re-SNB) procedure was performed.

**Results:**

Positive regional lymph nodes in recurrent breast cancer indicate a poorer outcome with axillary recurrences being the most favorable tumor site among all nodal regions. Most preferred staging method is ultrasound ± guided biopsy. PET-CT, scintimammography, SPECT-CT may improve visualization of affected lymph nodes outside the axilla. Concerning operative management 30 articles on re-SNB were identified with a mean harvesting rate of 66.4%, aberrant drainage and aberrant metastasis in 1/3 of the cases. Total rate of metastasis is 17.9%. After previous axillary dissection (ALND) the re-SNB has a significantly lower harvesting rate and higher aberrant drainage and aberrant metastasis rate. The prognostic outcome after re-SNB has been favorable.

**Conclusion:**

Nodal status in recurrent disease has prognostic value. The choice of operative management of clinically inapparent regional lymph nodes during local recurrence should be based on the previous nodal staging method. Patients with previous ALND should be spared a second systematic ALND. Re-SNB or no axillary surgery at all are possible alternatives. Lymphoscintigraphy may be performed to identify extraaxillary drainage. However, for definite recommendations randomized controlled studies are heavily needed.

**Supplementary Information:**

The online version contains supplementary material available at 10.1007/s00404-021-06352-9.

## Introduction

Breast Cancer is a common oncological disease characterized by a favorable prognosis due to its early detection and multimodal treatment options. Although the diagnostics and therapy of early breast cancer is standardized, there is a more challenging task with recurrent disease. Therapy options during breast cancer relapse are diverse and may vary depending on a cancer institute’s individual beliefs and experience. This is especially evident in the management of clinically negative lymph nodes during the reoperation. There is the possibility to perform an axillary node dissection (ALND), a (re-)sentinel node biopsy (SNB), nodal sampling or to not do anything at all. Due to the heterogeneity, we try to find answers to the following three central questions on the management of lymph nodes in recurrent breast cancer:

1. Which prognostic role does the lymph node status play in recurrent breast cancer?
2. What are useful staging methods for lymph node involvement?
3. Which surgical approach should be chosen with clinically negative lymph nodes?

With this review, we aim to give an overview of the extensive literature which might facilitate the clinical practice on recurrent breast cancer disease.

## Materials and methods

To assess the current recommended standard of care for breast cancer relapse, we studied the following national and international guidelines and recommendations:

- the German interdisciplinary S3-guideline on “Screening, diagnostic, therapy, follow-up-care of breast cancer (Früherkennung, Diagnostik, Therapie und Nachsorge des Mammakarzinoms), extended version 4.3, Feb, 2020”
- the recommendations of AGO, the German gynecologic-oncologic working group on “Loco-regional Recurrence”, version March, 2021.
- American NCCN guidelines Version 1.2021 Breast Cancer

**Fig. 1:**
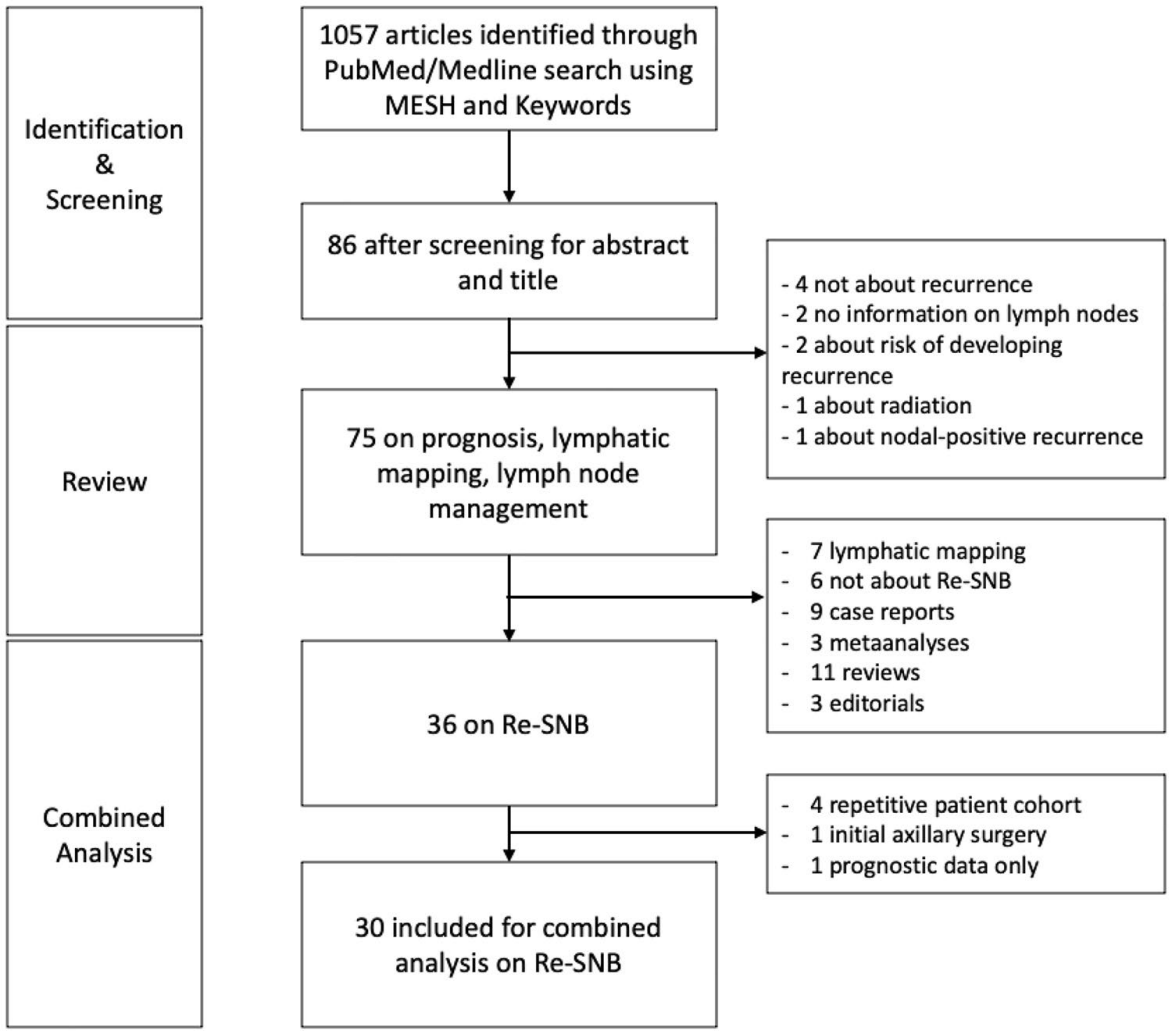
Flowchart of search strategy for systematic review

Furthermore, we conducted a structured search of the literature in PubMed using Medical Subject Headings (MeSH) and textwords. The search was conducted on Feb 1, 2021 and limited to studies published between Jan 1, 1999 and Feb 1, 2021. For details of the search strategy, see Fig. 1 and supplementary document 1.

We conducted a review of the literature on the feasibility and outcome of a Re-SNB in recurrent disease. For statistical analysis, descriptive statistics (percentage, mean, range) and *t* tests were performed.

## Results

### Prognostic role of lymph node status in recurrent disease

There are few known prognostic factors which may predict the disease progression and survival after breast cancer recurrence. The German S3-Guideline include a positive nodal status at primary diagnosis but not at recurrent disease as a poor prognostic factor. Lymphovascular space invasion is considered a poor prognostic factor for a second recurrence according to the AGO recommendations.

After exploring the literature on the prognostic role of nodal stage in recurrent disease, we identified articles which compared ipsilateral local breast tissue recurrence without lymph node or distant metastases (IBTR) to regional recurrence with lymphonodal involvement.

#### Overall, disease-free and distant disease-free survival

Compared to IBTR, regional recurrence in the lymph nodes is associated with worse overall, disease-free and distant disease-free survival. Anderson and al. analyzed 419 patients with recurrent disease, of which 342 patients had an IBTR and 77 patients had a locoregional recurrence either in the regional lymph nodes, chest wall or nonbreast skin. The 5-year overall survival and distant disease-free survival after a locoregional recurrence (34.9 and 27.8%, respectively) was considerably worse than after an IBTR (76.6 and 67.1%, respectively) irrespective of the initial therapy [1]. Another study by Harris et al. showed that the 5- and 10-year overall survival rates for patients with a regional lymph node recurrence (72 and 43%, respectively) was lower than for patients without any regional lymph node recurrence (91 and 81%, respectively) [2]. Montagna et al. analysed 197 patients with local recurrence in the ipsilateral breast or chest wall and 82 patients with regional lymphonodal recurrence with a median follow-up of 5.9 years and found a significantly higher risk of distant metastasis for patients with regional lymphonodal recurrence occuring more than 2 years after the primary diagnosis (Hazard ratio [HR] = 2.76; 95% CI 1.31–5.85) [3]. Lee et al. showed similar results. In 104 patients with ipsilateral locoregional recurrence, 35 patients (33.7%) had a regional lymph node recurrence. Compared to recurrent disease limited to the breast or chest wall, recurrent disease in regional lymph nodes was identified as an independent and significant prognostic factor for distant metastasis during a median follow-up of 8.9 years (HR of 2.816 (95% CI: 1.342–5.908, *p*: 0.006)) [4].

Interestingly, the impact of locoregional recurrence on survival seems to vary depending on the breast cancer subtype. Anderson et al. showed that patients with Estrogen-receptor(ER)-negative regional recurrence in the regional lymph nodes, chest wall or non-breast skin had a HR for mortality of 19.84 (95% CI: 13.33–29.54) compared to patients with ER-negative IBTR with a HR of 4.49 (3.29–6.13) [1]. In the study of Montagna et al., triple-negative disease in locoregional recurrence was associated with a higher risk of a subsequent relapse (HR 2.87 [1.67–4.91]) and death (HR 2.00 [1.25–3.19]) compared to other breast cancer subtypes.

#### Axillary lymph node recurrence

Although the results above seem consistent, their interpretation still poses difficulties. In many studies regional disease is summarized as multiple tumor localizations without the separate analysis of a single recurrence localization, e.g., the axillary lymph nodes, the most common nodal relapse site. In the study of Montagna et al. regional recurrence is defined as cancer in the axillary, internal mammary, supraclavicular or infraclavicular lymph nodes [3]. Lee et al. and Anderson et al. additionally count cervical lymph nodes and ipsilateral chest wall/non breast skin, respectively, to regional disease [1, 4].

In one study by Harris et al. isolated axillary lymph node recurrence were analyzed in comparison to other localizations. The author reports that over 50% of regional recurrence occurred in the axilla only. Axillary recurrence was shown as a favorable prognostic factor (*p* = 0.004) as it was associated with better overall, cause-specific and distant disease-free survival [2]. Similar results were reported in older studies by Fowble et al. and Recht et al. demonstrating that axillary lymph nodes were the most favorable recurrent site in terms of prognosis compared to other regional lymph nodes [5, 6]. In a study of the National Surgical Adjuvant Breast and Bowel Project (NSABP) trial, 424 patients with recurrent breast cancer and initially node-positive disease were analyzed, with 259 patients having a IBTR and 165 having a regional recurrence in the lymph nodes, chest wall or nonbreast skin. Patients with recurrence in the axillary nodes showed a higher 5-year distant disease-free survival rate (31.5%) compared to patients with supraclavicular metastasis (12.1%), but a lower disease-free survival rate compared to patients with IBTR (51.4%) [7].

### Staging methods for lymph node involvement in recurrent breast cancer

#### Ultrasound ± guided biopsy

In general, the most preferred screening and detection method for axillary lymph node metastases is an ultrasound followed by an ultrasound-guided biopsy.

According to a pooled analysis which included 9.232 cases of axillary staging procedures, 50% of axillary metastases were detected by an axillary ultrasound ± biopsy with a false-negative rate of 25% [8]. Another metanalysis with 31 publications found a higher accuracy for detection of axillary node involvement: a pooled sensitivity and specificity of 61.4 and 82% with ultrasound alone and 79.6 and 98.3% with the addition of ultrasound-guided biopsy [9]. Median positive predictive value (PPV) and negative predictive value (NPV) for the biopsy were estimated as 100 and 67.4%, respectively. According to Schipper et al. axillary ultrasound could detect advanced axillary disease (pN2-pN3) with a NPV of up to 97.7%, but could not accurately predict the difference between pN1 and pN2–pN3 disease [10].

Likewise, in recurrent breast cancer disease, ultrasound has been deemed useful in the detection of axillary and supraclavicular lymph node recurrences with a sensitivity, specificity and NPV of 76.9, 98.7 and 99.5%, respectively [11].

#### Magnetic resonance imaging (MRI)

In routine diagnostics, breast MRI can add supplementary information on the evaluation of locoregional tumor extent as well as the differentiation between scarring and recurrent lesions. In terms of nodal disease, mean sensitivity and specificity was 90% according to a meta-analysis which included 309 patients. MRIs enhanced with ultrasmall super-paramagnetic iron oxide performed slightly better with a mean sensitivity and specificity of 98 and 96%, respectively [12]. When comparing MRI to an axillary ultrasound, the NPV for the detection of advanced axillary disease was slightly higher for MRI (99.3 vs. 98.5%). In addition, the differentiation between pN1 and pN2-pN3 disease was more accurate by MRI, which leads to the conclusion that axillary ultrasound may be omitted in case the breast-MRI shows no pathological nodal findings [13].

#### Computer tomography (CT)

A CT scan is routinely performed as a staging tool for distant metastases. Although it is not primarily designated for nodal surveillance, lymph node recurrences can be detected using CT imaging. DeSelm et al. mapped the anatomic pattern of isolated nodal recurrences with axial CT scans and identified the axilla as the most frequent site of nodal recurrence with 42%. Other regional recurrence sites include the internal mammary and the supraclavicular region with 32.5 and 25.5%, respectively [14]. In recurrent disease, Abi-Sheisha et al. showed that CT could detect lymph node metastases > 1 cm easily, but might be inferior to PET-CT in the detection of smaller and extra-axillary nodal recurrences [15].

#### Fluordesoxyglucose Positron-Emission Tomography (FDG-)PET-CT

PET-CT is usually not included in routine breast cancer staging, though it might be helpful when an area of interest is not furtherly accessible by conventional imaging or biopsy.

For the general detection of axillary lymph node involvement at primary disease, PET-CT might be inferior to conventional imaging with a sensitivity up to 70% compared to 85.7%. However, it has a high specificity with up to 100%. Furthermore, PET-CT proves itself superior to conventional methods when detecting extra-axillary lymph node involvement [16, 17]. This feature might especially be useful at breast cancer relapse, as PET-CT has displayed a higher distribution of metastases in the internal mammary and supraclavicular region than at primary disease [18]. In recurrent setting, PET-CT showed much higher detection rates for axillary and extra-axillary lymph node involvements compared to CT and whole-body MRI with a diagnostic accuracy of up to 98.7 vs. 77.5% and 75%, respectively [19, 20].

#### Scintimammography

*S*cintimammographies are performed using the injection of radioactive tracers (e.g., Technetium-99 m sestamibi) in the opposite arm of the suspected cancer lesion. After 5–10 min, planar scintigraphs are taken with a gamma camera which visualizes the thoracal field including the breast, chest wall and axillary region. This method is not recommended in routine breast cancer screening and staging but can be performed supplementary in selected cases, such as in patients with dense breast tissue or palpable masses without mammographic or sonographic findings.

For axillary staging, scintimammography (sensitivity: 64%, specificity: 90%) performed better than mammography but worse than ultrasound (sensitivity: 73%, specificity: 95%) [21]. In recurrent setting though, the sensitivity of scintimammographies in detecting regional lymph node metastases was much higher with 93% [22].

#### Operative staging, lymphoscintigraphy, SPECT/CT

Several studies have shown that an operative staging via sentinel lymph node biopsy has a higher sensitivity in detecting lymph node metastases compared to PET or MRI [12, 23]. Sentinel lymph nodes are usually localized using blue-dye or, nowadays more commonly, radiocolloid infection and a planar lymphoscintigraphy. In case the visualization fails, a SPECT/CT may be added. In a study with 134 patients, SPECT/CT discovered 19 additional sentinel lymph nodes in 15 patients including 4 nodal metastases which otherwise would have been missed. An additional advantage of SPECT/CT was estimated in 42% of the patients through improved localization and more precise operative incision [24]. Another study with 741 patients showed that SPECT/CT was able to detect 97.8% of sentinels and was not restricted to Level I of the axillary region only but localized sentinels in Level II, III, the internal mammary chain and supraclavicular region [25]. Borelli et al. analyzed the role of SPECT/CT in breast cancer relapse. Compared to planar lymphoscintigraphy, SPECT/CT showed a slightly higher visualization rate of sentinel nodes (53.3 vs. 43.4%, nonsignificant) with 19 additionally detected nodes. Except for one case, all nodal metastases were found in basins outside the ipsilateral axilla.

### Surgical management of clinically negative lymph nodes in recurrent disease

#### Guidelines and recommendations

After previous ALND, both the German S3 Guideline and AGO recommendations don’t recommend any axillary intervention in case of clinically negative lymph nodes.

For women who underwent previous SNB, the consensus recommendation of the NCCN is the performance of an ALND of Level I and II. A Re-SNB should be avoided after previous mastectomy, since its prognostic significance is unknown, but may be considered after previous lumpectomy. The German AGO recommendation classifies a re-SNB as a potential “disadvantage for patients” and that “it might not be performed”. In case the re-SNB procedure is performed nevertheless but no sentinel lymph node is detected, an ALND as well as an operative intervention outside the ipsilateral axilla should not be performed.

### Literature research, summarized results

After extensive literature research we identified 30 articles with 1945 cases on the feasibility and outcome of the Re-SNB procedure in recurrent disease. There were 5 articles analyzing patients with previous sentinel node biopsy only, 2 on previous ALND only, 14 on breast-conserving therapy only and 1 on mastectomy only. 13 articles analyzed patients with mixed characteristics. All identified literature is listed in Table 1. Its combined results are summarized in Table 2.

**Table 1:** 30 articles included in the pooled analysis

Author	Patients (previous ALND/SNB)	Technique	Sentinel node (SLN) harvesting rate	Abberant drainage	Metastases	Operative management	Follow-up (FUP)
Sato [50]	*n* = 136 - ALND: 55 - SNB: 69 - no surgery: 12 BCS: 100%	Radiocolloid (Tc99m-phytate)	87% (118/136) ALND: 80% SNB/no surgery: 91% *p* < 0.001	49% (58/118) ALND: 75% SNB: 33% - most common: IM / contra	n.A	n.A	n.A
Sávolt Á [33]	*n* = 160 - ALND: 80 - SNB: 80 BCS: 100% Radiation: 95%	Radiocolloid (technetium-99 m) ± Blue dye	66% (106/160) - ALND: 55% - SNB: 78% *p* < 0.003	24,5% (26/106) - ALND: 43% - SNB: 11% *p* < 0.001	9% (9/106): - 6 Micro - 3 Macro 7 Ipsi 2 contra	- contra: SNB - parasternal/IM: no operation - Micro: no ALND - Macro: ALND - Unsucessful Re-SNB: ALND or axillary revision	n.A
Biglia [41]	*n* = 71 - ALND: 26 - SNB: 33 - no axillary surgery: 12	n.A	75% (53/71) - ALND: 54% - SNB / no surgery: 88% *p* = 0.009	13.2% (7/53)	10% (5/51) - 3 Micro - 2 Macro	- Macro: ALND - Unsucessful Re-SNB: 4/18 ALND (surgeon’s choice)	n.A
Karanlik [26]	n = 75 - ALND: 52 - SNB: 23 ––––––––– - BCS: 44 - ME: 31	Radiocolloid (technetium-99 m) ± Blue dye	56% (42/75) - ALND: 44% - SNB: 83% *p* = 0.002 –––––––––– - BCS: 71% - ME: 36% *p* = 0.003 Best detection rate: BCS + SNB: 86%	64% (27/42) - ALND: 91% - SNB: 32% *p* = 0.001 ––––––––––- - BCS: 58% - ME: 82% *p* = 0.16	35,9% (14/39) - 6 Micro - 8 Macro ––––––––– SNB: 86% Ipsi 29% IM ALND: 14% Ipsi 71%, IM 42.9% contra	Micro: ALND Macro: ALND	Mean FUP: 36 m No locoregional recurrence
Matsumoto [28]	*n* = 35 ALND: 8 SNB: 22 No surgery: 5 –––––––––- BCS: 100% Radiation: 68,6%	Radiocolloid (Technetium-99 m) + Blue dye ± ICG (in case of failure)	80% (28/35) - ALND: 75% - SNB: 81.8% - no surgery: 80% *p* = 0.52	14% (4/28) - ALND: 37.5% - SNB: 4.5% - no surgery: 0%	7% (2/28) - 1 macro ipsilateral (SLN) - 1 micro contralateral (ALND)	Macro: ALND Micro: no ALND	Median FUP: 40,3 m No axillary recurrence
Folli [36]	*n* = 30 SNB: 100% (-) BCS: 100%	Radiocolloid (Technetium-99 m)	90% (27/30)	11% (3/27) - 2 IM - 1 SCV/ICV	13% (4/30) - 2 Re-SLNs - 1 non-SLN - 1 Unsucessful Re-SNB	All patients: back-up ALND Unsuccessful Re-SNB: ALND	NPV: 95.2%, Acurracy: 95.6% False negative rate:33%
SNARB: Vugts [35] Poodt [51] Poodt [44] Poodt [45]	*n* = 536 ALND: 288 SNB: 214 No surgery: 34 BCS: 375 ME: 61	Radiocolloid (Tc99m) + Blue Dye	53% (287/536) - ALND: 43% - SNB: 64% - no surgery: 78%	54% (180/333) - ALND: 82% - SLB: 28% *p* < 0.001	20% (56/287) - 10 ITC - 17 Micro - 29 Macro - 2 false-negative Re-SLN Extraaxillary (contra, IM): 84,6% ALND 25% no ALND	ALND: Pos. Re-SLN: 9 Neg. Re-SLN: 31 Unsuccessful Re-SNB: 60/239 Previous ALND: 40	NPV: 93,6% Neg. Re-SLN without additional ALND: Median FUP: 4,7 y Regional recurrence: 4,5% (9/201), 7/9 aberrant basins Unsuccesful Re-SLN: 5-y-regional-recurrence: ALND: 0%, no axill. surgery: 3,7% p = 0.113 All patients, median FUP: 5.1 y 3,5% regional recurrence - 1/18 pos. Re-SLN - 10/18: neg. Re-SLN - 7/18: unsuccesfull Re-SLN (p = 0.618)
Uth [37]	*n* = 147 - ALND: 74 - SNB: 73 - BCS: 133 - ME: 14	Radiocolloid (Technetium-99 m) or / and Blue Dye	50% (72/144) - ALND: 34% - SNB: 66% *p* = 0.0001 - BCS: 48% - ME: 64% *p* = 0.25	17% (12/72) - ALND: 25% - SNB: 13% *p* = 0.18 - BCS: 44% - ME: 13% *p* = 0.018	24% (17/72) - 11 Micro - 6 Macro - 14/17 Ipsi (5 ALND, 9 SNB) - 3/17 abberant (ALND: 1 contra axilla, SNB: 2 intrapectoral/ IM 20% (5/25) unsuccessful Re-SNB	ALND: Negative Re-SLN: 8/72 Unsuccessful Re-SNB: 25/72	False-negative rate: 0% Median FUP: 38 m No axillary recurrence
Intra [52]	*n* = 212 - SNB: 100% (-) - BCS: 100%	Radiocolloid (Technetium-99 m) 1-day prior	93% (196/212)	8% (17/207) - 6 contra - 4 intramammary - 4 interpectoral - 2 IM - 1 ICV	16% (34/196) - 25 Macro - 9 Micro/ITC 0 metastases in aberrant drainage	Aberrant drainage: surgically removed only when accessible Macro: ALND Micro/ITC: no ALND Unsuccessful Re-SLN: ALND only in high-risk patients	Median FUP: 48 m - 5-y-LR: 8,3% - 5y-axillary recurrence: 3,9% 5-year DFS: 79.9% 5-year OS: 93.9%
Tokmak [34]	*n* = 7 ALND: 2 SNB: 5 BCS: 100%	Radiocolloid (Technetium sulfur) ± Blue Dye Same day	86% (6/7) ALND: 100% SNB: 80%	50% (3/6) - 2 contra (1 ALND, 1 SNB) - 1 IM (ALND)	17% (1/6) - 1 micro (ALND, contra)	Micro: ALND (contra) Unsucessful SNB: ALND	Median FUP: 27 m No lymph node recurrences
Kaur [29]	*n* = 45 ALND: 100% BCS: 100%	Radiocoolid (Technetium sulfur) or / and Blue Dye	29% (13/45)	39% (5/13) - 3 contra - 3 IM All previous radiation	39% (5/13) - 1 contra - 1 IM	Unsucessful SNB: no ALND	Median FUP: 21,6 m No lymph node recurrences
Derkx [40]	*N* = 13 SNB: 100% (-) - BCS: 12 - ME: 1	Radiocolloid or/ and Blue Dye	39% (5/13)	n.A	18% (2/11) - 1 Re-SLN - 1 unsuccessful Re-SNB	ALND - Pos. Re-SLN: 1 - Neg. Re-SLN: 1 - unsuccessful Re-SNB: 6	Median FUP: 13 m no evidence of disease
Tasevksi [53]	*n* = 18 - ALND: 15 - SNB: 3 - BCS: 16 - ME: 2	Radioisotope ± Blue Dye	67% (12/18) - ALND: 67% - SNB: 67%	75% (9/12) - 5 contra - 5 IM All previous ALND	25% (3/12) - 1 contra - 2 ITC (1 contra, 1 IM)	contra: ALND ITCs: no ALND	n.A
Karam [42]	*n* = 20 - ALND: 11 - SNB: 4 - no axillary surgery: 5 ME: 100%	Radioisotope (Tc99m-Sulfur) ± Blue Dye	65% (13/20) - ALND: 46% - SNB: 100% - no surgery: 80% p = 0.106	13% (2/15) - 1 IM - 1 SCV	25% (4/16) - 2/13 Re-SLN (ALND/no surgery) - 2/3 unsucessful Re-SNB No aberrant metastases	ALND: Pos. Re-SLN: all Neg. Re-SLN: 2/11 Unsucessful Re-SNB and no previous ALND: 3/7	Mean FUP: 33.3 m 2 re-recurrences with neg. Re-SLN: - metaplastic carcinoma, metastases 3 months later - 2^nd^ chest-wall + axillary recurrence, 5-year disease free
Cox [43]	*n* = 56 SNB (−): 100%	Radioisotope (Tc99m-sulfur) and/or Blue Dye	80% (45/56)	2% (1/45) - 1 IM	21% (10/47) - 9 pos. Re-SLN - 1 unsuccessful Re-SNB	ALND: Pos. Re-SLN: all Unsuccessful Re-SNB and invasive cancer: 2/11	Mean FUP: 26 m - no axillary recurrences - 1 death (unknown cause)
Schrenk [31]	*n* = 30 - ALND: 13 - SNB/node Sampling: 15 - no surgery: 2	Radioisotope (Tc99m) + Blue Dye	63% (19/30) - 0–3 nodes removed: 82% - > 10 nodes removed: 39% *p* = 0.02	32% (6/19) ALND: 60% SNB: 14% *p* < 0.0001 - 4 IM - 1 interpectoral - 1 contra	16.7% (5/30) - 3 pos. Re-SLN - 1 ITC (Level III) - 1/11 unsuccessful Re-SNB	ALND: Pos. Re-SLN: all except internal mammary Neg. Re-SLN: 13/15 Unsuccessful Re-SNB: - 3/11 ALND (1 pos.) - 8/11 axillary exploration	False negative rate: 0%
Axelsson [54]	*n* = 47 - ALND: 43 - SNB: 1 - no surgery: 3 - BCS: 33 - ME: 14	Radioisotope (Tc99m) ± Blue Dye	45% (20/44)	15% (7/47) - 1 contra - 2 SCV - 3 parasternal - 2 close to recurrent site	35% (7/20)	Most patients: ALND, but not all	n.A
Barone [55]	*n* = 19 - ALND: 12 - SNB: 7 BCS: 100%	Radioisotope ± Blue Dye	84% (16/19) - ALND: 75% - SNB: 100% p: n.s	n.A	13% (2/16) - both after previous ALND	Pos. Re-SLN: ALND	Mean FUP: 15 m No axillary recurrence
Intra [32]	*n* = 65 BCS: 100% SNB: 100% (-)	Radioisotope (Tc99m)	97% (63/65)	8% (5/63) - 4 IM - 1 SCV	11% (8/65) - 7 pos. Re-SLN - 1 unsuccessful Re-SNB	ALND: Pos. Re-SLN: 6/7 Unsuccessful Re-SNB: 2/2 No ALND: Pos. Re-SLN in IM: 1/7	Median FUP: 45,9 m 57 women without ALND: - no axillary recurrence - 2 local recurrence - 1 new primary - 1 metastases
Koizumi [30]	*n* = 31 - ALND: 16 - SNB: 3 - no surgery: 12 BCS: 100%	Radioisotope (Tc99m-phytate)	90% (28/31) - ALND: 81% - no ALND: 100%	45% (13/29) - 5 IM (all ALND) - 7 contra (all radiation)	14% (4/28) - 2 IM - 1 contra	n.A	n.A
Port [38]	*n* = 117 - ALND: 63 - SNB: 54 BCS: 100%	Radioisotope (Tc99m-sulfur) + Blue Dye	55% (64/117) - ALND: 38% - SNB: 74% *P* = 0.0002	30% (19/63) - 11 IM - 1 SCV - 1 ICV - 3 Intra - 5 Contra 84% ALND	18,8% (12/64) - 2 IM (ALND) - 2 non-SLN: intra, ipsi	ALND Pos. Re-SLN: all Neg. Re-SLN: 23/54 Back-up Unsuccessful Re-SNB: some	False-negative rate 17% Median FUP: 2,2 y - no axillary recurrences - 6 systemic disease without local recurrence
Roumen [27]	*n* = 12 - ALND: 10 - SNB: 2 - BCS: 10 - ME: 2	Radioisotope (Tc99m) + Blue Dye	83% (10/12) - ALND: 80% - SNB: 100% - BCS: 81,8% - ME: 100%	64% (7/11) - 3 IM - 4 contra All ALND	50% (5/10) - 2 contra, 1 ipsi + parasternal (ALND)	contra: axillary exploration	Median FUP: 24 m Negative Re-SLN: no recurrences
Boughey [56]	*n* = 21 - ALND: 12 - SNB: 5 - No surgery: 4 - BCS: 17 - ME: 4	Radioisotope (Tc99m) and/ or Blue Dye	61% (13/21) - ALND: 50% - no ALND: 78% - BCS: 65% - ME: 50%	46% (6/13) - 3 IM - 2 contra > 10 LN removed: 50%	7.7% (1/13)	Unsuccessful Re-SNB: some ALND	Median FUP: 13 m No axillary recurrences
Taback [57]	*n* = 15 - ALND 9 - SNB: 6 BCS: 100%	Radioisotope (Tc99m-sulfur) + Blue Dye	79% (11/14) - ALND: 67% - SNB: 83%	63% (7/11) - 1 SCV - 2 IM - 2 interpectoral - 3 contra	27% (3/11) - 2 contra	ALND: Pos. Re-SLN: 2/3 Neg. Re-SLN: 7/8 Unsuccessful Re-SNB: ¾	Median FUP: 41 m - 80% no evidence of disease - 2 deaths (metastases): 1 pos. Re-SLN, 1 neg. Re-SLN - 1 (lymphoma)
Newman [58]	*n* = 10 - ALND: 7 - SNB: 1 - No surgery: 2 BCS: 100%	Radioisotope (Tc99m-sulfur) + Blue Dye	90% (9/10) ALND/no surgery: 100% SNB: 0%	78% (7/9) - 3 IM - 7 contra All ALND	0%	n.A	n.A
Dinan [59]	*n* = 16 - ALND: 14 - SNB: 2	Radioisotope (Tc99m-sulfur) ± Blue Dye	69% (11/16) ALND: 64% SNB: 100%	64% (7/11) - 2 SCV - 1 IM - 3 contra Axilla - 1 contra SCV	0%	ALND: Neg. Re-SLN: 1 Unsuccessful Re-SNB: 1	n.A
Sood [60]	*n* = 4 ALND: 100% BCS/no breast surgery: 100%	Radioisotope (Tc99m-sulfur) + Blue Dye	100% (4/4)	100% (4/4) - 2 IM - 1 contra - 2 intra	0%	N.A	N.A

**Table 2:** Summarized results of 30 articles

	Total	Previous SNB	Previous ALND	*p* value
Sentinel node harvesting rate	66.4% (1291/1945)	79.8% (693/869)	49.0% (400/816)	0.009
Aberrant drainage	32.6% (442/1357)	16.5% (120/726)	81.8% (318/389)	< 0.001
Aberrant metastasis	33.7% (58/171)	13.1% (11/84)	64.3% (45/70)	< 0.001
Total metastasis	17.9% (218/1220)			

#### Success of re-sentinel node biopsy

The mean harvesting rate of re-sentinel nodes was 66.4% (1291/1945). The success was significantly higher in patients with previous SNB (79.8%, range: 38.5–100%) compared to patients with previous ALND (49.0%, range: 29–100%, *p* = 0.009). When comparing previous breast-conserving surgery (BCS) and mastectomy (ME), the sentinel node harvesting rate was similar (66.5% (1059/1593) vs. 64.2 (86/134), *p* = 0.29); however, it should be noted, that far less cases with previous mastectomy were investigated. When collectively evaluating the previous operative procedure of breast and axilla, the combination of initial BCS and SNB yielded the best success rates for re-SNB at recurrence [26, 27].

#### Aberrant drainage

Re-SNB was performed using a Technetium-99 m-based radiocolloid, blue dye or both. In one case, indocyanine green (ICG) fluorescence was added when the conventional method failed and identified additional nodes [28]. Lymphatic drainage was visualized by preoperative lymphoscintigraphy or located intraoperatively using a gamma probe. Aberrant drainage was seen in 32.6% of cases, with a significantly higher rate after previous ALND (81,8%) compared to previous SNB (16.5%, *p* < 0.001). Most common regional drainage sites outside the ipsilateral axilla were: internal mammary, supra-/infraclaviculary and interpectoral region as well as the contralateral axilla. Aside from axillary dissection, previous radiation seemed to influence the drainage pattern as well. In two studies all patients with lymphatic drainage to the contralateral axilla had a history of radiation during primary disease [29, 30].

#### Metastases

Lymph node metastases were found in 218 out of 1220 cases (17.9%). In articles which specified metastatic sites, a third were found in aberrant basins (33.7%). Patients with previous ALND were more likely to have positive nodes outside the ipsilateral axilla than patients with previous SNB (64.3 vs. 13.1%, *p* < 0.001). Most common aberrant metastatic sites were the internal mammary and contralateral axillary region.

#### Operative management

When a positive macrometastatic sentinel node was detected, a complementary—sometimes contralateral—ALND or axillary exploration was performed. In few cases, when the positive node was found in the internal mammary region only, no additional axillary operation was performed [31–33]. Similarly, the detection of micrometastases or isolated tumor cells (ITC) were usually not followed by ALND with few exceptions [26, 34].

When a negative sentinel node was found. a ALND was usually considered unnecessary. However, many studies including the SNARB-study with the largest number of cases (*n* = 536) performed back-up ALNDs to evaluate the false-negative rate of the re-SNB procedure [31, 35–38]. According to a meta-analysis which included 170 patients with back-up ALND after negative re-SNB, the overall false-negative rate was low (9.4%). The sensitivity, negative predictive value and the accuracy of the re-SNB procedure were 90,6%,95,9% and 97,1%, respectively [39].

When a sentinel node was not visualized or detected, the operative management was heterogenous across all studies. Some studies performed ALND in all patients with unsuccessful re-SNB [31, 33, 34, 36, 40], others left the decision to the surgeon [38, 41]. In some cases, an ALND was performed depending on the previous axillary operation [35, 42]. Kaur et al. did not perform ALND at all, as all patients had previous ALND at primary disease [29]. Intra et al. performed ALND in high-risk patients and Cox et al. in patients with invasive recurrent disease [32, 43]. Uth et al. did not specify their reason for performing or omitting an ALND, but found metastases in 20% (5/25) of patients with ALND after unsuccessful SNB.

#### Outcome and prognosis

The prognosis after Re-Sentinel biopsy has been favorable. In many studies which omitted an ALND in case of negative Re-sentinels, no axillary recurrence has been observed after a median follow-up time up of 27 months (range: 15–46,9 months). (Karanlik, Matsumoto, Tokmak, Kaur, Cox, Barone, Intra, Roumen).

As the largest study on Re-SNB, the SNARB-study furtherly explored its prognostic value. After a median follow-up of 5.1 years, regional recurrence was observed in 18 patients (3.5%). 1 out of 18 patients had a positive Re-sentinel node, 10 had a negative Re-sentinel node und 7 had unsuccessful Re-SNB. Distant recurrences occurred in 88 patients (17.1%). The 5-year distant disease-free survival in the patients with negative, positive and unsuccessful re-SNB was comparable with 85.4, 76 and 84.7%, respectively. In conclusion, the SNARB-study found no significant association between the outcome of Re-SNB and the occurrence of regional (*p* = 0.618) and distant recurrence (*p* = 0.682) [44]. Moreover, according to their results, an ALND after an unsuccessful Re-SNB may be omitted, as the risk of developing a regional recurrence is negligible irrespective of a followed ALND (5-year regional recurrence rate: 0% for ALND vs. 3.7% for no surgery, *p* = 0.113) [45]. However, it has been noted that when additionally considering the initial axillary operation, the majority of the unsuccessful Re-SNB group had an ALND, either at prior disease or at recurrence. Ultimately out of 239 cases with unsuccessful Re-SNB, only 26 patients were spared an ALND [46]. The low number of cases makes the validity of the results questionable.

In contrast to the SNARB study, prior axillary staging method did not matter in a study by Ugras et al. In this study, all patients with an ipsilateral breast or chest wall recurrence had a previous negative SNB. Out of 83 patients which were included, 47 were treated with a re-SNB and 36 without axillary surgery. The outcome after a median follow-up of 4.2 years was comparable between both groups. Axillary and non-axillary recurrence as well as death rate were low with 2.6, 5.5% and 3.9%, respectively. Distant metastasis occurred in 8 patients (12.5%) with 5 in the re-SNB and 3 in the non-surgery group. Thus, this study demonstrates the feasibility of the Re-SNB procedure but questions its worth and value [47].

## Discussion

### Prognostic role of nodal status in recurrent breast cancer disease

Although the guidelines don’t explicitly list nodal status in recurrent disease as a prognostic factor, there is evidence that a positive nodal involvement might be associated with poorer survival. Axillary node recurrences seem to have a worse prognosis compared to local ipsilateral breast disease but are a more favorable tumor site compared to other nodal regions.

### Staging methods for lymph node involvement

The most preferred imaging method for detection of lymph node metastases is an ultrasound ± ultrasound-guided biopsy. This method is fast, cost-effective, uses no radiation and has a high sensitivity and specificity at primary disease and at recurrence. An MRI might be supplementary added. CTs, though not intended for nodal screening specifically, might detect enlarged lymph nodes during routine staging for distant metastasis.

In recurrent disease, lymph node metastases occur more frequently in extraaxillary basins due to aberrant drainage after initial breast cancer therapy. Supplementary nuclear medical imaging such as PET-CT or scintimammography may improve visualization of affected lymph nodes outside the axilla during relapse. For sentinel node biopsy, adding a SPECT-CT to planar lymphoscintigraphy increases the detection rate of sentinels, thus reducing the possibility of overlooking nodal metastases.

### Operative management

To summarize the literature on the operative management of clinically inapparent regional lymph nodes during breast cancer relapse, the following conclusions can be made: The choice of operation should be based on the axillary staging method at initial breast cancer disease. Patients with previous ALND should be spared a second systematic ALND as suggested by the German S3 guidelines and AGO recommendations. Lymphoscintigraphy may be performed to identify extraaxillary drainage basins.

Patients with previous SNB may receive a Re-SNB. This procedure has been proven as a feasible alternative option especially after initial BCS. In case of no visualization of the Re-sentinel node, omitting a secondary ALND may also be safe. In the study by Kaur et al. patients with unsuccessful Re-SNB did not receive a secondary ALND. At a median follow-up of 21.6 months, there were no lymph node recurrences [29]. In the SNARB study, the majority (75%) of the patients with unsuccessful Re-SNB were spared an ALND. Yet the prognostic outcome was comparable to patients who received an ALND [45].

When mapping the Re-sentinel node, failure of lymphatic drainage was greatly associated with previous ALND and radiation as shown by the studies above. Both procedures are potentially damaging to lymphatic vessels. Whenever there is no sign of radioactive or blue dye migration from the breast to the adjacent regions, is it safe to assume that cancer cells in the breast are also not able to spread lymphatically, since the lymphatics are likely disrupted? The scenario of breast cancer cells being “trapped” locally may further encourage the omission of an ALND when the Re-sentinel node can’t be visualized.

Another question concerns the rate of regional lymph node metastases and its prognostic value. As seen in the combined results above, the rate of metastases was as high as 17,9%. However, in the SNARB study, the histological result of the re-SNB (benign, malign or unknown) did not influence the prognosis of the patients [44]. Does this mean, that an operative axillary staging including a Re-SNB can be completely abandoned, since its outcome did not matter? Are clinical and radiographic staging sufficient as long as they are inapparent? In the study by Ugras et al. there was no difference in the prognosis of patients whether they received a Re-SNB or no axillary surgery at all [47].

A possible explanation is that, rather than the radical surgical removal of regional nodes, the use of adjuvant therapy may have a larger prognostic impact in the recurrent setting of breast cancer. This has been demonstrated in the CALOR trial. Patients with breast cancer recurrence treated with adjuvant chemotherapy had a significantly longer disease-free survival and breast-cancer free interval compared to patients without chemotherapy. In a multivariable proportional hazards model, the location of the recurrence (i.e. in the regional lymph nodes) did not affect the patient’s outcome, whereas the interval to first surgery and tumor subtype were identified as independent prognostic factors [48].

Furthermore, considering the historical development of surgical breast cancer treatment, it has been proven that doing more is not always better. In the last decades, the operating techniques had shifted from radical mastectomies and ALNDs for all patients to breast-conserving surgery and SNB whenever the clinical indication allows. This resulted in a nowadays substantially lower morbidity in breast cancer surgery without risking a higher mortality. One of the most burdensome treatment-related conditions is the development of lymphedemas. It heavily impairs quality of life, since there is only symptomatic treatment but no cure. The risk of developing lymphedemas is significantly higher after ALND than SNB [49]. Especially at breast cancer recurrence, when scarring in the lymphatics has been formed due to former operation and sometimes additional radiation, there may be an even increased risk of lymphedema development when performing additional axillary surgery. With this aspect in mind, the choice of the lymph node staging method and its radicality should be chosen even more cautiously in the recurrent setting.

In the end, uncertainties remain as all literature on the axillary staging procedure using the Re-SNB in recurrent breast cancer are retrospective analyses. As of now, there is no published randomized controlled study which compares the outcome of recurrent breast cancer patients after a Re-SNB vs. an ALND vs. no axillary surgery. To give definite recommendations for the clinical decision-making, this specific question should be further explored through prospective studies in the future.

## Supplementary Information

Below is the link to the electronic supplementary material.

Supplementary file1 (DOCX 19 KB)

## Data Availability

Literature review in Pubmed, AGO recommendations, NCCN guidelines.
