# Supplementary material for: Prognostic value and management of regional lymph nodes in locoregional breast cancer recurrence: a systematic review of the literature
Source: Arch Gynecol Obstet. 2022 Feb 4;306(4):943–57. doi: 10.1007/s00404-021-06352-9 (PMC9470629; doi:10.1007/s00404-021-06352-9)
Supplement: Supplementary file 1 — Supplementary file1 (DOCX 19 KB) [file 404_2021_6352_MOESM1_ESM.docx]

**Supplementary document 1:**

To cover subjects of breast cancer recurrence, staging and management of the axilla, we used the following keywords.

**breast [tiab] AND (cancer [tiab] OR neoplas*[tiab] OR tumor[tiab])**

**OR**

**Breast Neoplasm [MH:NoExp] OR Inflammatory Breast Neoplasms [MH] OR Triple Negative Breast Neoplasms[MH] OR Carcinoma, Ductal, Breast[MH]**

**AND**

**local recurrence [tiab] OR relapse [tiab] OR recurrent [tiab] OR ipsilateral recurrence [tiab] OR isolated local recurrence [tiab] OR chest wall recurrence [tiab]**

**AND**

**axillary lymph node dissection [tiab] OR lymph node removal [MH] OR lymphadenectomy [tiab] OR axilla* [MH] OR sentinel biopsy [MH] OR axillary surgery [MH] OR axillary procedures [MH] OR sentinel node [MH]**

We identified 1057 articles using the keywords and MESH terms above. After screening for title and abstract, 86 articles remained, of which 11 were excluded as they were not about the subject of interest. 75 records on prognostic value, lymph node mapping and operative management were included for the preparation and composition of the systematic review of the literature. For the combined analysis, 7 records on lymphatic mapping only, 6 without data on Re-SNB and 26 non-original articles were excluded. Out of 36 articles, 4 articles were about studies which had been updated with larger patient cohorts in more recent published articles (SNARB, Intra et al. (2005)[1], Port et al. (2002)[2]) and thus were excluded. In one article, patients with recurrences didn’t have any previous axillary surgery at all and in one article, only prognostic data on Re-SNB were available.

**References**

1. Intra M, Trifirò G, Viale G, Rotmensz N, Gentilini OD, Soteldo J, Galimberti V, Veronesi P, Luini A, Paganelli G, Veronesi U. Second biopsy of axillary sentinel lymph node for reappearing breast cancer after previous sentinel lymph node biopsy. Annals of surgical oncology. 2005;12(11):895-9. doi: 10.1245/aso.2005.10.018.

2. Port ER, Fey J, Gemignani ML, Heerdt AS, Montgomery LL, Petrek JA, Sacchini V, Van Zee KJ, Borgen PI, Cody HS, 3rd. Reoperative sentinel lymph node biopsy: a new option for patients with primary or locally recurrent breast carcinoma. Journal of the American College of Surgeons. 2002;195(2):167-72. doi: 10.1016/s1072-7515(02)01268-1.
